# The Shutdown Dissociation Scale (Shut-D)

**DOI:** 10.3402/ejpt.v6.25652

**Published:** 2015-05-13

**Authors:** Inga Schalinski, Maggie Schauer, Thomas Elbert

**Affiliations:** Department of Psychology, University of Konstanz, Konstanz, Germany

**Keywords:** Shutdown dissociation, assessment, PTSD, multiple trauma, subtype

## Abstract

The evolutionary model of the defense cascade by Schauer and Elbert (2010) provides a theoretical frame for a short interview to assess problems underlying and leading to the dissociative subtype of posttraumatic stress disorder. Based on known characteristics of the defense stages “fright,” “flag,” and “faint,” we designed a structured interview to assess the vulnerability for the respective types of dissociation. Most of the scales that assess dissociative phenomena are designed as self-report questionnaires. Their items are usually selected based on more heuristic considerations rather than a theoretical model and thus include anything from minor dissociative experiences to major pathological dissociation. The shutdown dissociation scale (Shut-D) was applied in several studies in patients with a history of multiple traumatic events and different disorders that have been shown previously to be prone to symptoms of dissociation. The goal of the present investigation was to obtain psychometric characteristics of the Shut-D (including factor structure, internal consistency, retest reliability, predictive, convergent and criterion-related concurrent validity).

A total population of 225 patients and 68 healthy controls were accessed. Shut-D appears to have sufficient internal reliability, excellent retest reliability, high convergent validity, and satisfactory predictive validity, while the summed score of the scale reliably separates patients with exposure to trauma (in different diagnostic groups) from healthy controls.

The Shut-D is a brief structured interview for assessing the vulnerability to dissociate as a consequence of exposure to traumatic stressors. The scale demonstrates high-quality psychometric properties and may be useful for researchers and clinicians in assessing shutdown dissociation as well as in predicting the risk of dissociative responding.

Based on the defense cascade model, we developed the Shutdown Dissociation Scale (Shut-D; Schauer & Elbert, 2010), which is able to meet the new requirements for assessing the expression of derealization and depersonalization as a consequence of shutting down emotions, sensations, cognitions, and as a result behavior that would be suboptimal for surviving certain threats. The DSM-5 defines a dissociative subtype of posttraumatic stress disorder (PTSD) recognizing that those patients exhibit additional symptoms of primarily depersonalization and derealization (Friedman, Resick, Bryant, & Brewin, 2011; Lanius, Brand, Vermetten, Frewen, & Spiegel, 2012). To support the subtype hypothesis, it is necessary to describe the symptom profiles and differentiating factors. More research is needed to clarify whether dissociative symptoms occur in a distinct PTSD subgroup with a high symptom severity and distinct neurobiological profile or whether there is a dissociative continuum within the PTSD (Dalenberg & Carlon, 2012). Similar to the concept of shutdown dissociation, the DSM-5 committee links the dissociation to an overwhelming experience that may arise when the individual is confronted with an overwhelming threat with perceived inescapability, such as childhood sexual abuse, torture, or war trauma (American Psychiatric Association, 2013). Being confronted with an imminent life-threat, for which flight-or-fight is no longer a viable option to counter danger, the organism may shift to immobility and dissociative responding. To escape the threatening situation as well as the internal distress and arousal, dissociative responding may be adaptive. The defense cascade model by Schauer and Elbert (2010) considers the corresponding shutdown dissociation as a progression on the defense cascade that enhances survival (Bracha, 2004; Bradley, Codispoti, Cuthbert, & Lang, 2001; Lang, Bradley, & Cuthbert, 1998; Table 1).

**Table 1:** Assumed survival advantage of the shutdown continuum according to Schauer and Elbert (2010) in order to inhibit non-adaptive action disposition and enable survival

Freeze	During attentive immobility (orienting response) - blend in with its surroundings by remaining as motionless as an inanimate object - shift the attention of predators to other moving or noisy stimuli
Flight/fight	During active defense response - increase in heart rate, blood pressure, faster and deeper breathing - increase in sweating (cools the body, moist palms at the same time allow for a better grip in order to flee, lowering the chances for injury) - the release of sympathetically mediated adrenalin is initiated - the heart and muscles with the required energy for flight or fight - peripheral vessels constrict in order to reduce potential blood loss in the case of an injury - shutdown of perception for nociception to inhibit recuperative behavior - limitation of cognitive ability
Fright	During unresponsive immobility - increase in survival chances even when physical contact has been made, because movement cues are critical, releasing stimuli for predatory behavior - immobility helps avoiding tissue damage when threatened with sharp objects or when penetrated - immobility signals surrender and eliminates cues for counter aggression - shut down/numbing of anger emotion to inhibit aggression and defense - suppressed vocal behavior
Flag/faint including flaccid immobility	During flaccid immobility - lowering blood pressure in case of tissue damage, minimizing blood loss - “automatic” shock-bedding – availability of oxygen and nutrients in central organs (i.e., the brain) - decrease in heart rate while maintaining metabolism in case of intoxication or contamination - cardio-protection (→ cortisol stops stress reaction) - analgesia - numbing of all emotions including fear, disgust, etc. - shutdown of physiological arousal and memory functions

## The defense cascade model

In life-threatening situations, the ongoing perceptual and behavioral processes would initially be interrupted, followed by enhanced sensory perception towards the threatening stimulus (Graham & Clifton, 1966; Sokolov, 1963). If the stimulus is threatening, then the sympathetic branch of the autonomous nervous system becomes dominant and the release of sympathetic mediated adrenalin is initiated. This bodily adaption supplies the heart and muscles with the required energy for flight-or-fight. At the same time, amongst a concert of other actions, the peripheral vessels constrict in order to reduce potential blood loss in the case of injury. In life-threat with extreme fear, an immediate flight-or-fight response may not be optimal and thus “fright” or tonic immobility is common (Bracha, 2004; Porges, 1997). Reports about tonic immobility from rape survivors describe similar states in humans (e.g., Galliano, Noble, Travis, & Puechl, 1993). Further stages of the defense repertoire include “flag–faint” with its dissociative properties. Maximal proximity of danger, such as penile penetration during rape, is associated with more dissociative responding (Johnson, Pike, & Chard, 2001). It consists of functional sensory deafferentation, motor paralysis, alterations of the consciousness, and loss of speech perception and production. To shut down the bodily system, the parasympathetic system takes over dominance, resulting in bradycardia, a decrease in blood pressure, and vasodilatation (Scaer, 2001). “Fright–flag–faint” becomes adaptive when there is no perceived possibility for flight-or-fight.” Dissociative responding may be conditioned (Bolles & Fanselow, 1980). First, disruption of the ongoing perceptual or bodily experiences provides the basis for shutdown dissociation and interferes with an integrative representation of the environment and the self (Schauer & Elbert, 2010). It is likely that this ongoing disruption of integrative processes would play a key role in the development and maintenance of PTSD. Dissociative responding could then be understood, on the one hand, as an adaption in order to survive during life-threat and, on the other hand, as a problem as resulting in more fragmentation of the past and future memories.

The item generation of the Shut-D was based on patients’ symptom descriptions, clinical observations, and expert judgment, and was conceptualized on the basis of the outlined defense cascade model. The scale has been applied in several studies (Fiess, Steffen, Pietrek, & Rockstroh, 2013; Isele et al., 2014; Schalinski, Elbert, & Schauer, 2011, 2013; Schalinski, Moran, Schauer, & Elbert, 2014) that have shown the close relationship between trauma-related psychopathology and shutdown dissociation.

## Measurements of dissociation in patients

One of the first studies about dissociation in PTSD applied the Dissociative Experiences Scale (DES; Bernstein & Putnam, 1986). This is a self-rating scale that contains normal and pathological dissociative states and was developed as a tool to assess dissociation in the general population. The authors of the scale referred to the concept of a dissociative continuum that ranges from minor dissociative experiences to major pathological dissociation, such as the multiple personality disorder. The Clinician-Administrated Dissociative State Scale consists of both a self-rating and ratings scored by a professional observer (Bremner et al., 1998). Furthermore, there exist structured interviews for assessing diagnostic criteria of dissociative symptoms such as Structured Clinical Interview for DSM-IV (Steinberg, 1994) and Dissociative Disorder Interview Schedule (Ross et al., 1989). The Multidimensional Inventory of Dissociation is a 218-item self-administered instrument, especially for clinical research and diagnostic assessment (Dell, 2006). Other types of self-rating questionnaires that measure dissociative responding include the Multiscale Dissociation Inventory (Briere, Weathers, & Runtz, 2005) and the Somatoform Dissociation Questionnaire (SDQ-20; Nijenhuis, 2001; Nijenhuis, Spinhoven, Van Dyck, Van Der Hart, & Vanderlinden, 1996). The SDQ-20 measures somatoform dissociation that is closely related to Janet's concept of dissociation and includes positive (e.g., pain perception) and negative symptoms of dissociation (e.g., loss or reduction of acoustic perception; Nijenhuis, Vanderlinden, & Spinhoven, 1998; Van Der Hart, Nijenhuis, Steele, & Brown, 2004). Phenomenologically, there are similarities between items of the SDQ-20 and the Shut-D. The fifth version of the Diagnostic and Statistical Manual of Mental Disorders (DSM-5) describes the PTSD Dissociative Subtype with prominent symptoms of alternated (usually integrated) functions of consciousness, memory, sense of time, body awareness, and perceptions of the environment and the self (American Psychiatric Association, 2013). The development of instruments has been based on clinical observations rather than on a biological model of dissociative responding. The item construction of the Shut-D has been based on biological dimensions and the neural system that is systematically shut down (Schauer & Elbert, 2010). Shutdown dissociation includes partial or complete functional sensory deafferentiation, classified as negative dissociative symptoms (see Nijenhuis, 2014; Van Der Hart et al., 2004). The Shut-D focuses exclusively on symptoms according to the evolutionary-based concept of shutdown dissociative responding. The perspective of the defense cascade model offers a conceptual framework for research on psychopathology of dissociation across diagnostic entities and a clinically valid proposal with new treatment strategies to counterstrike adverse effects of shutdown dissociation (a list of therapeutic techniques has been provided by Schauer & Elbert, 2010). The items are scored in an interview to be applicable also in resource-poor settings (because self-ratings require well-educated, literate respondents). The newly developed scale should help to systematically record the impact of traumatic experiences with high proximity to danger and serve as a research tool for shutdown dissociative responding. The goal of the present investigation was to obtain psychometric characteristics (factor structure; internal consistency; retest reliability; and predictive-, convergent-, and criterion-referred concurrent validity) of the Shut-D. Different patient samples were selected that have been prone to symptoms of dissociation in previous reports such as patients with psychotic spectrum disorders, major depression, borderline personality disorder, PTSD, and dissociative identity disorder (e.g., Putnam et al., 1996).

## Methods

### The Shutdown Dissociation Scale (Shut-D)

The Shut-D is a structured interview consisting of 13 items. Responses to all items were given on a scale including 0 (not at all), 1 (once a week or less), 2 (2–4 times a week), to 3 (5 or more times a week). Summed scores can range from 0 to 39. When completing this interview, interviewers should establish the time frame for which these shutdown dissociation symptoms have been reported. The interviewer should select a time frame within the past 6 months in order to acquire an overview of the patient's suffering in their everyday life. If the trauma occurred less than 6 months ago, symptoms are to be explored since the traumatic event.

### Administration and scoring rules

1. Use the prompt questions as written on the questionnaire; use additional questions as needed to accurately determine the frequency of the symptom.
2. Use open-ended questions to carefully inquire about the frequency. When was the last time you suffered from this symptom? When you think back over the last month, was [the symptom] a rare occurrence? Have you only sometimes experienced this symptom or does it occur often? What did/does it mean for you?
3. It is appropriate to use information that arises later in the interview to modify an earlier rating.
4. If a person reports that he or she experiences spells of fainting, the interviewer should rate all corresponding symptoms measured by the scale (e.g., acoustic, visual, and motor as well as pain perceptual shutdown).
5. Ask questions that are useful in distinguishing between a shutdown/defensive symptom and an acute or chronic medical condition or peripheral neuropathy:
  - How long have you been suffering from this symptom?
  - Shutdown dissociation simulates central nervous system neuropathy. Peripheral neuropathy describes the damage to the peripheral nervous system. Peripheral damage affects one or more dermatomes and thus produces symptoms for specific areas of the body. In contrast, shutdown dissociation affects a part of the body (e.g., the whole hand and the whole leg) or the whole body.
6. Please consider side effects of medication, and exclude if it was due to effects of alcohol or drugs.
7. Please consider similar effects that may appear during adolescence or at the beginning of menopause.

### Subjects and demographical data

#### Study sample 1

We recruited female refugees with multiple traumatic experiences at the University of Konstanz outpatient clinic for refugees. They were referred to the clinic by a human rights organization, medical doctors, or lawyers for diagnostic clarification or potential treatment. All patients participated in the assessment of shutdown dissociation. Complete data were obtained from 54 patients and 17 healthy controls with similar ethnic backgrounds, who were recruited from the general community. Following this, the number of traumatic experiences was assessed using the sum of the event checklist of the Clinician Administered PTSD Scale (Blake et al., 1995). For traumatic events, we made a distinction between the number of traumatic event types that were self-experienced and the number of traumatic event types that were witnessed. A traumatic event type was judged as self-experienced if the participant was the victim (high proximity of danger), or a witness (low proximity of danger) if the participant had observed the traumatic event while someone else was threatened. For PTSD diagnosis, we used the Clinician Administered PTSD Scale and summed its score for symptom severity. The score on the Hamilton Rating Scale for Depression (Williams, 1988) estimated the degree of depression. Out of the patient group, 42 fulfilled all criteria of the diagnosis; two fulfilled the DSM-IV criteria of PTSD (A, B and D, E, F) but met only two of three avoidance symptoms (criteria C); whereas the ten others fulfilled the criteria for depression and subclinical PTSD symptoms. Data of the same sample are presented in Schalinski et al. (2013, 2014).

#### Study sample 2

The study sample 2 consisted of German psychiatric patients and healthy controls (Table 2). The level of dissociation was assessed using the Shut-D and the DES (Bernstein & Putnam, 1986). The responsible psychologist or the psychiatrists in charge made the current diagnoses based on the International Classification of Mental and Behavioral Disorders Tenth Version (ICD-10; World Health Organization, 1992).

**Table 2:** Sample description, mean, and standard deviation of age, frequency of gender, and mean and standard deviation of shutdown dissociation

Sample	Age, *M* (*SD*)	Gender, % female	Shutdown Dissociation Score, *M* (*SD*)
Study sample 1 (*n*=71) HC (*n*=17) MD (*n*=10) PTSD (*n*=44)	32 (6.8) 30 (13.7) 35 (10.1)	100	8 0.94 (1.25) 4.8 (3.4) 17 (8.6)
Study sample 2 (*n*=77) HC (*n*=51) BPD (*n*=13) MD (*n*=13)	31.5 (11.2) 30.4 (10.3) 27.6 (8.7) 42.2 (14.5)	100	2.02 (3.1) 14.88 (10.7) 5.77 (4.7)
Study sample 3 (*n*=130) PSD (*n*=104) BPD/MD (*n*=26)	29.2 (9.1) 26.5 (6.1)	38 33.7 53.8	4.3 (4.7) 8.03 (5.8)
Study sample 4 DID (*n*=15)	43 (9.1)	100	19.2 (9.4)

#### Study sample 3

Patients (*n*=130) were recruited from the inpatient pool at the local Psychiatry in Germany. The sample included 104 patients with a diagnosis of psychotic spectrum disorder and 26 patients with borderline personality disorder or/and major depression. In this sample, we screened for adverse childhood experiences and applied the Shut-D. Childhood adversities were recorded using the Maltreatment and Abuse Chronology of Exposure (MACE) scale for adults (Isele et al., 2014; Teicher & Parigger, 2015), which was specially developed to retrospectively capture the exposure to multiple childhood adversities up to the age of 18. We used the MACE SUM score that indicates the overall severity of exposure. The responsible psychologist or the psychiatrists in charge made the current diagnoses based on the ICD-10 (World Health Organization, 1992), and verified that the patient had sufficiently improved to provide informed consent and could participate in the assessment of adverse childhood experiences. The inclusion criteria were at least age 18, and receiving treatment at the local psychiatry in the post-acute treatment section. Thus, the patient sample consisted of patients that were motivated for further treatment. Two patients refused to participate for the following reasons: one because the participant felt bothered by his childhood experiences and one participant because of distrust.

#### Study sample 4^1^

This sample consisted of 15 female patients with dissociative identity disorder (Schlumpf et al., 2013). According to Schlumpf and colleagues, patients were recruited from private practitioners and psychiatric outpatient departments in Switzerland and Germany for an fMRI study for biosocial reaction. Exclusion criteria were comorbid psychotic disorder, drug abuse or addiction, antisocial or histrionic personality disorder, and a neurological or organic brain disease. The clinical diagnosis was additionally confirmed by clinical experts in dissociative disorders using the German version of the Structured Clinical Interview for DSM-IV Dissociative Disorders (Gast, Oswald, Zündorf, & Hofmann, 2000). The average age of the sample was *M*=43 (*SD*=9.1). During data collection, the patients were requested to answer the items for their main host personality. The level of dissociation was assessed using the Shut-D and the DES.

### Statistical and data analysis

Analyses were performed using R version 2.15.1 and SPSS 21 with an alpha level of 5%. The alpha level was set at 0.1% for multiple group comparisons using Bonferroni adjustment (see Criterion-referenced concurrent validity section). The factor structure as well as the internal consistency and item-total correlation were assessed in all study samples. The test–retest was used to assess the reliability of the scale in the whole study sample 1. Furthermore, predictive validity was investigated in a symptom provocation paradigm (study sample 1; see Predictive validity section), convergent validity was examined between the Shut-D and the DES (study samples 2 and 4), criterion-referenced concurrent validity was obtained by comparisons of different diagnostic groups (study samples 1–4) and point-biserial correlates with the symptom spectrums depression and PTSD symptom severity (study sample 1). To avoid global correlations, the associations were assessed in the patient sample.

### Predictive validity

Participants were exposed to rapid visual serial presentation of emotionally arousing and neutral pictures from the International-Affective-Picture-System (Lang, Bradley, & Cuthbert, 2008). Following exposure, a psychologist interviewed the participants about their shutdown dissociative responding. The tendency towards shutdown dissociation was rated on a Likert scale with possible scores of 0 (not at all), 1 (a little bit), 2 (moderately), 3 (strongly), and 4 (very strongly) during picture presentation using the 13-item Shut-D Intensity Scale. Although the unpleasant pictures were not personalized for the traumatic events, 60% of the PTSD sample experienced intrusive memories of their own trauma that were triggered by the stimulation (Schalinski et al., 2014).

## Results

### Reliability

#### Internal consistency

We performed a principal axis factoring analysis using the Kaiser–Guttman criterion to determine the factor structure in the data of the samples 1–4 (*n*=293). The first factor (eigenvalue 5.65) accounted for 43.43% of the variance, whereas the second factor (eigenvalue 1.07) accounted for 8.19% of variance. All other eigenvalues were below 1. Table 3 presents the factor loadings of the items as well as the rotated factor solution (Varimax procedure). The internal consistency of the scale was examined with Cronbach's α. The questionnaire showed excellent internal consistency with Cronbach's α=0.89 in its original item composition. The internal consistency could not be improved through item deletion.

**Table 3:** Item difficulties and factor loadings in an one-factor solution as well as in a rotated (Varimax) two-factor solution

			One-factor solution	Two-factor solution^a^	
				
	Items	*M* (*SD*)	1. Factor loading (43.43%)	1. Factor loading (43.43%)^a^, (27.25%)^b^	2. Factor loading (8.19%)^a^, (24.37%)^b^
1	Have you fainted?/Have you been passing out?	0.2 (0.58)	0.58	0.24	0.60
2	Have you felt dizzy and has your vision gone black?/Felt dizzy and couldn't see anymore, as though you were blind?	0.83 (1.06)	0.72	0.57	0.45
3	Have you felt as though you couldn't hear for a while, as though you were deaf? When people were talking to you, did they sound far away?	0.49 (0.96)	0.68	0.21	0.78
4	Have you had an experience of not being able to properly see things around you (e.g., blurred vision)	0.62 (1.0)	0.71	0.37	0.65
5	Have you felt as though your body or a part of your body has gone numb?	0.64 (1.08)	0.71	0.51	0.50
6	Have you felt as though you couldn't move for a while, as though you were paralyzed?	0.37 (0.81)	0.72	0.71	0.28
7	Have you felt as though your body, or a part of it was insensitive to pain (analgesia)?	0.46 (0.93)	0.64	0.33	0.58
8	Have you been in a state in which your body suddenly felt heavy and tired?	1.0 (1.32)	0.60	0.61	0.23
9	Have you experienced that your body becoming stiff for a while?	0.39 (0.85)	0.64	0.80	0.08
10	Have you felt nauseous? Have you felt as though you were about to throw up? Have you felt yourself break out in a cold sweat?	0.80 (1.10)	0.62	0.68	0.19
11	Have you had an “out-of-body” sensation? Have you felt as though you were outside of your body?	0.43 (0.90)	0.50	0.06	0.68
12	Have you had moments in which you have found yourself unable to speak?/Have you been able to speak only with great effort?/Have you had an experience in which you could only whisper for a period of time?	0.46 (0.91)	0.70	0.63	0.35
13	Have you felt suddenly weak and warm?	0.48 (0.95)	0.71	0.51	0.50

*Note*.

a Before rotation

b rotated (Varimax) factor matrix.

#### Item-total correlation

The item-total correlation was performed in the whole sample. All Shut-D item-to-total correlations were significant at *p<*0.001. Item-total correlations ranged from *r*_s_ *=*0.44 (item 1: Have you fainted?/Have you been passing out?) to *r*_s_=0.72 (item 2: Have you felt dizzy and has your vision gone black?/felt dizzy and couldn't see anymore, as though you were blind?). These consistently moderated to strong associations, indicating that every item was correlated with the sum score (Table 4).

**Table 4:** Item-total-correlation as well as retest reliability index on item level

	Item	Item-total correlation	Retest reliability index on item level^a^
1	Fainting	0.44, *p*<0.001	0.73, *p*<0.001
2	Dizziness/transitory blindness	0.72, *p*<0.001	0.74, *p*<0.001
3	Transitory deafness, changed acoustic perception	0.62, *p*<0.001	0.68, *p*<0.001
4	Changed visual perception	0.64, *p*<0.001	0.76, *p*<0.001
5	Numbness	0.67, *p*<0.001	0.66, *p*<0.001
6	Transitory paralysis	0.56, *p*<0.001	0.59, *p*<0.001
7	Analgesia	0.55, *p*<0.001	0.76, *p*<0.001
8	Heavy and tired	0.68, *p*<0.001	0.66, *p*<0.001
9	Tension	0.56, *p*<0.001	0.38, *p*=0.007
10	Feeling of nausea	0.68, *p*<0.001	0.67, *p*<0.001
11	Out of body	0.45, *p*<0.001	0.82, *p*<0.001
12	Inability to speak	0.60, *p*<0.001	0.87, *p*<0.001
13	Weakness and hot flash	0.62, *p*<0.001	0.70, *p*<0.001

*Note*.

a The retest reliability index on item level was calculated in study sample 1.

#### Test–retest reliability

**Fig. 1:**
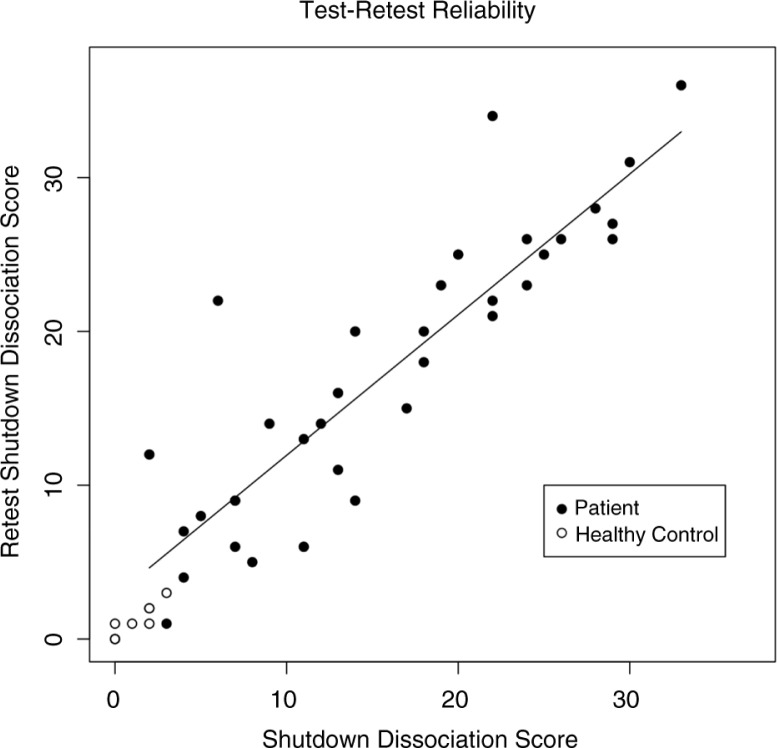
The relationship between the sum score of the first and second assessments of the shutdown dissociation score. The filled circles present members of the patient group of the study sample 1 (patients with posttraumatic stress disorder and/or major depression) and the open circles members of the healthy control group. The line indicates the regression line (model estimation from the patient sample).

The study sample consisted of 50 participants (38 patients with PTSD and/or depressive disorders according to the criteria of the DSM-IV) and 17 healthy controls. The mean shutdown dissociation score at the first assessment was *M*=15.1 (*SD*=9), and *M*=17.14 (*SD*=9.13) at the second assessment, whereas the healthy control group reported significantly lower scores upon the first (*M*=0.83, *SD*=1.17) and second assessments (*M*=0.81, *SD*=1.03). The length of the test–retest interval was on average *M*=39 days and *SD*=27 (range 7 and 134 days). The test–retest reliability index was high (*r*=0.93, *p*<0.001, CI_95_=0.88–0.96). The test–retest reliability was 0.87. Figure 1 shows the correlation between the first and second assessments. On a single item level, the test–retest reliability ranged from *r*_s_ *=*0.38 (item 9) to *r*_s_ *=*0.87 (item 12), reaching a significance level of *p*=0.007 (item 9), and all other *p*<0.001 (Table 4).

### Validity

#### Predictive validity

**Fig. 2:**
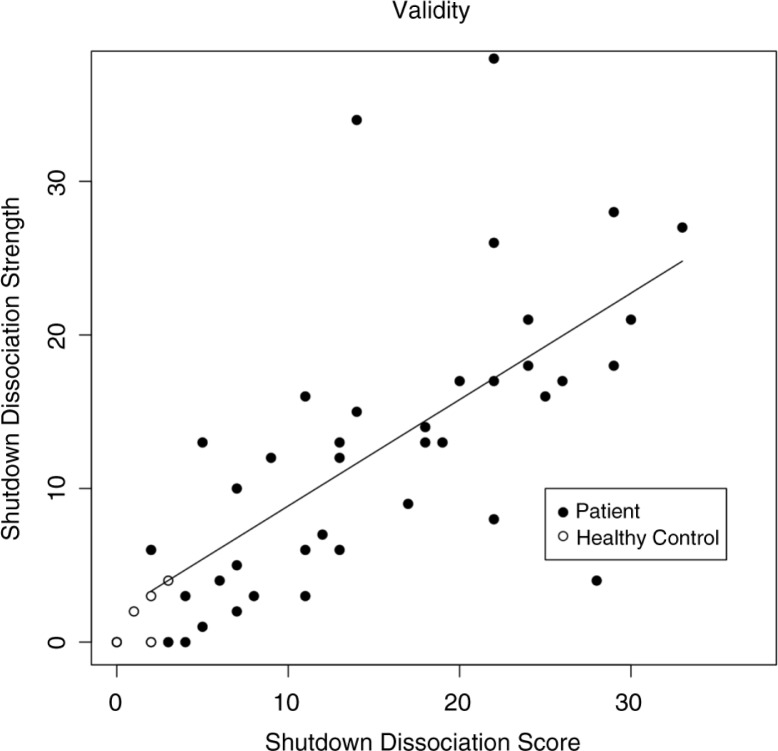
The relationship between the shutdown dissociation score and the shutdown dissociative strength in response to emotional evocative pictures. The filled circles present a PTSD/MD group and the open circles a non-PTSD group member. The line shows the regression line (model estimation from the patient sample). PTSD=posttraumatic stress disorder, MD=major depression.

The shutdown dissociation strength in response to rapidly presented pictures was assessed in a study designed to trigger trauma-specific processing (Schalinski et al., 2014). Those patients with PTSD or trauma-related depressive symptoms (*n=*40) who displayed high Shut-D scores in their daily lives also reported elevated shutdown dissociation during the exposure of emotional evocative pictures (*r=*0.66 and *p<*0.001). The correlation was higher when the healthy control group (*n*=17) was considered (*r=*0.79 and *p<*0.001). The scatterplot is presented in Fig. 2.

#### Convergent validity

**Fig. 3:**
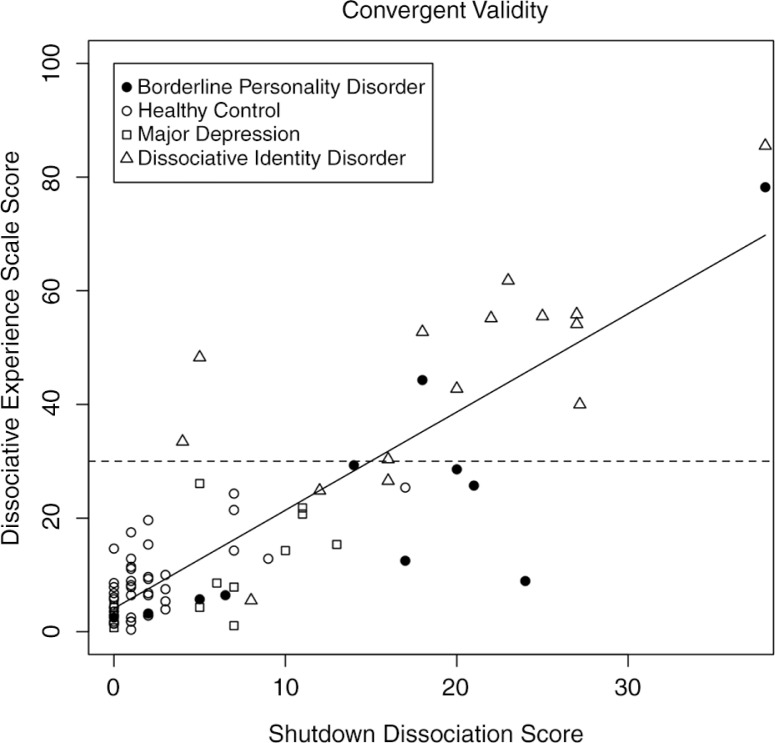
The scatterplot of the shutdown dissociation sum score and the sum score of the Dissociative Experience Scale (DES) across different diagnostic groups and healthy controls. The patients in the dissociative identity disorder group were instructed to rate the symptoms for the host personality. The dashed horizontal line presents the cut-off score of the DES sum score, and those values above 30 are indicative of a dissociative disorder or of posttraumatic stress disorder (PTSD).

In a study of 10 female patients with borderline personality disorder, 12 patients with a diagnosis of depression, 15 patients with dissociative identity disorder, and 48 healthy controls, the convergent validity of the DES and the Shut-D was assessed (Fig. 3). The correlation of the sum scores was significant (*r*=0.86, *p*<0.001). The Shut-D showed significant associations with the subscales of the DES: amnesia *r*=0.70, *p*<0.001; absorption *r*=0.72, *p*<0.001; and derealization *r*=0.74, *p*<0.001.

#### Criterion-referenced concurrent validity

**Fig. 4:**
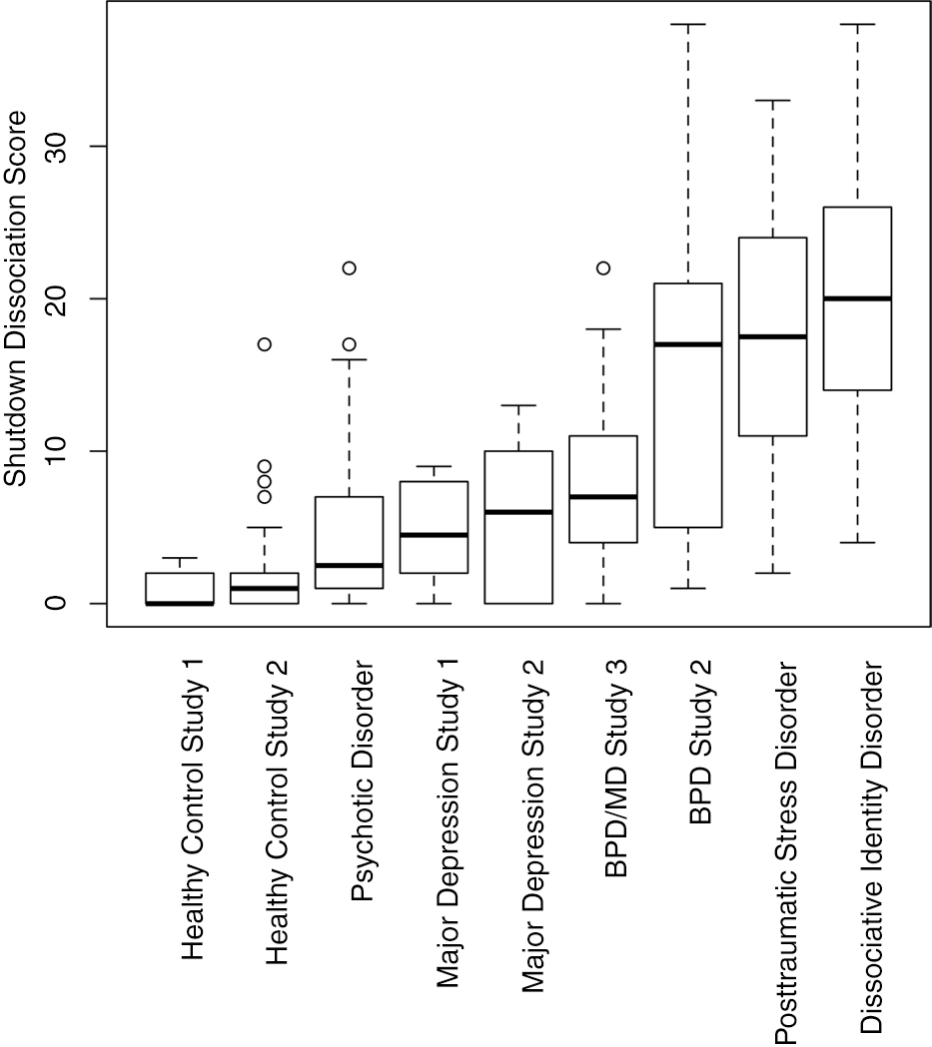
Boxplot of the shutdown dissociation sum score across different diagnostic groups and healthy controls. The patients in the dissociative identity disorder group were instructed to rate their symptoms for the host personality. BPD=borderline personality disorder, MD=major depression.

Figure 4 shows the sum score for different diagnostic groups. The diagnostic groups affected the Shut-D score significantly (*χ* ^2^_(8)_=133.26, *p*<0.001). In the study sample 1, all groups (healthy control, major depression, and PTSD) differed from one another in their Shut-D score (all pairwise Wilcoxon rank sum tests: *p*≤0.003). In the study samples 2 and 3, both the clinical groups (major depression and borderline personality disorder) showed higher Shut-D scores compared to the healthy control group (all *p*≤0.001). Furthermore, the patients with borderline personality disorder scored higher on the Shut-D compared to patients with psychotic spectrum disorders as well as to patients with major depression (all *p*<0.001). There was no significant difference between the Shut-D scores comparing groups of patients with PTSD and dissociative identity disorder (Fig. 4 and Table 5).

**Table 5:** Group comparisons between different diagnostic groups as well as healthy controls

Sample	Healthy control Study 2	Psychotic disorder	MD Study 1	MD Study 2	MD/BPD Study 3	BPD Study 2	PTSD	DID
Healthy Control Study 1	*W*=347 *p*=0.203	*W*=454 *p*=0.001*	*W*=28.5 *p*=0.003	*W*=48 *p*=0.006	*W*=44 *p*<0.001*	*W*=210 *p*<0.001*	*W*=734.5 *p*<0.001*	*W*=255 *p*<0.001*
Healthy Control Study 2		*W*=3,502 *p*=0.001*	*W*=130 *p*=0.013	*W*=200 *p*=0.025	*W*=217 *p*<0.001*	*W*=600.5 *p*<0.001*	*W*=2,152.5 *p*<0.001*	*W*=746 *p*<0.001*
Psychotic disorder			*W*=433 *p*=0.382	*W*=560 *p*=0.312	*W*=801 *p*=0.001	*W*=1,112.5 *p*<0.001*	*W=*4,110.5 *p*<0.001*	*W=*1,450 *p*<0.001*
MD Study 1				*W*=56 *p*=0.594	*W*=88.5 *p*=0.146	*W*=101.5 *p*=0.025	*W*=392.5 *p*<0.001*	*W=*137 *p*<0.001*
MD Study 2					*W*=206.5 *p*=0.268	*W*=600.5 *p*<0.001*	*W*=496.5 *p*<0.001*	*W*=173 *p*<0.001*
MD/BPD Study 3						*W*=232 *p*=0.062	*W*=911.5 *p*<0.001*	*W*=327 *p*<0.001*
BPD Study 2							*W*=333.5 *p*=0.371	*W=*125 *p*=0.213
PTSD								*W*=374 *p*=0.448

*Note*.

* Significant at Bonferroni adjusted *p*-value (*p*=0.001). MD=major depression, BPD=borderline personality disorder, PTSD=posttraumatic stress disorder, DID=dissociative identity disorder.

### Correlates of the Shut-D (event-type related)

**Fig. 5:**
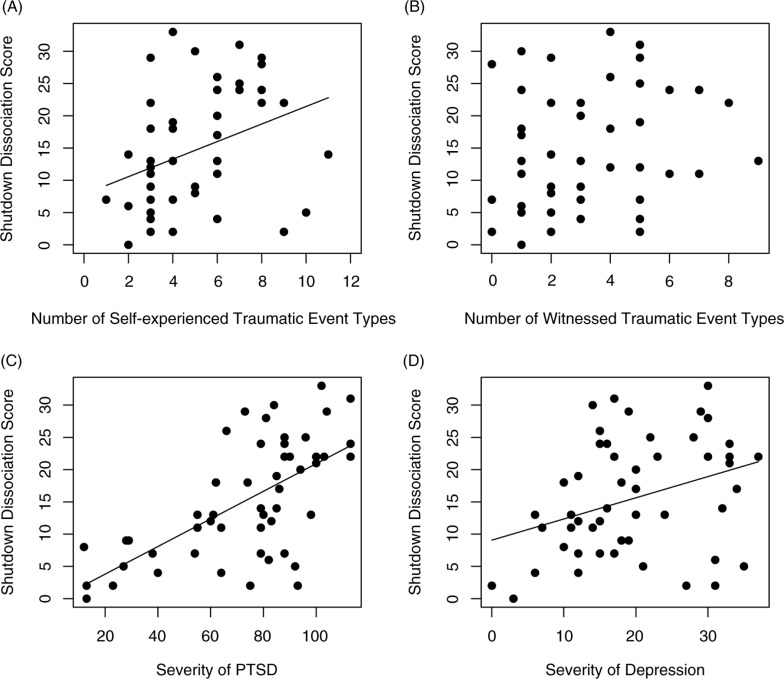
Scatterplots illustrating correlations between shutdown dissociation and (A) the number of different traumatic event types, (B) the number of different witnessed traumatic event types, (C) the severity of PTSD symptoms, and (D) the severity of depression symptoms. The slope of the regression is presented for significant associations. PTSD=posttraumatic stress disorder.

In study sample 1, the point-biserial correlation of the event type (1=experienced sexual assault with vaginal/oral/anal penetration; 0=no such experience) and the Shut-D score was significant (*r*=0.31, *p*=0.034, *n*=47). Furthermore, the Shut-D score was correlated with physical assault (1=experienced; 0=not experienced; *r*=0.31, *p*=0.042, *n*=52). The Shut-D was associated with the number of different event types that were self-experienced (high proximity of danger; *r*=0.37, *p=*0.007), but not with the number of traumatic event types that were witnessed (low proximity of danger; *r*=0.25, *p*=0.085; Fig. 5).

In study sample 3, the severity of childhood maltreatment (MACE SUM score) was positively related to the Shut-D (*r*=0.42, *p*<0.001). Especially, the emotional subscales of the MACE correlated with the severity of Shut-D (peer verbal abuse: *r*=0.38, *p*<0.001; parental non-verbal emotional abuse *r*=0.39, *p*<0.001; emotional neglect *r*=0.31, *p*<0.001; parental verbal abuse *r*=0.29, *p*=0.001). Furthermore, the Shut-D score was significant related to the severity of physical neglect (*r*=0.36, *p*<0.001).

### Symptom levels (PTSD and depression)

In the study sample 1 (*n*=53), the Shut-D was positively correlated with the severity of PTSD (*r=*0.67, *p*<0.001) and the Hamilton depression score (*r=*0.33, *p*=0.018; Fig. 5). Partial correlations were calculated to examine the common variances of these symptom clusters. Upon examining partial correlations between the PTSD symptom severity and the Shut-D score while controlling for the depression score, a significant positive correlation remained (*pr=*0.51, *p*<0.001). Although the correlation between depression and the Shut-D score was significant, the partial relationship (when considering the variation of the PTSD severity) was considerably diminished (*pr=*−0.01, *p*=0.973). When the influence of the Shut-D was partialled out, the correlation between the depression and PTSD severity was still positive (*pr=*0.45, *p*=0.001).

## Discussion

Within the context of a brief interview, the Shut-D scale assesses dissociative responding as a consequence of traumatic stress based on a psychobiological model of the defense cascade (Schauer & Elbert, 2010). The assessment is suitable for different levels of education and has been successfully applied in different samples, including low-income countries, migrant samples, and various psychiatric disorders (Fiess et al., 2013; Isele et al., 2014; Schalinski et al., 2011, 2013, 2014). This report shows high-quality psychometric characteristics for data collected from healthy controls, samples with PTSD, major depression, psychosis, borderline personality disorder, and dissociative identity disorder. Results demonstrated sufficient internal reliability and excellent test–retest reliability of the Shut-D. Furthermore, the scale shows high convergent validity with the sum score of the DES, a scale that has dominated in the research of dissociation in patients with PTSD. The Shut-D score reliably not only separates patients with exposure of trauma and psychopathology from healthy controls (with and without trauma exposure) but also differentiates between diagnostic groups that are associated with different amounts of trauma exposure. The scores of the diagnostic groups are consistent with the clinical expectation. Those disorders that are particularly related to trauma exposure, such as PTSD, borderline personality disorder, or dissociative identity disorder, show the highest scores (Briere, Hodges, & Godbout, 2010; Halligan, Michael, Clark, & Ehlers, 2003; Murray, Ehlers, & Mayou, 2002; Nijenhuis, 2001). The more different traumatic event types a person experienced, the more likely shutdown dissociation becomes the primary mode of physiological responding. The data suggest that the exposure to those traumatic experiences with a high proximity to danger (such as sexual and physical assaults) enhances the variety and frequency of shutdown dissociation alongside an escalating shutdown of bodily functions (sensory, emotional, and nociceptive perception) terminating in tonic or flaccid immobility. The shutdown dissociation model hypothesizes that symptoms of shutdown dissociation emerge in relation to high proximal exposure to threat, for example, sexual abuse. Thus, we found higher scores in respondents who reported substantial sexual abuse. The prevalence of reported abuse in individuals with Borderline Personality disorder ranges from 62% to 71% (e.g., Paris et al., 1994a, 1994b; Zanarini et al., 2002), and the reported abuse is even higher in those with dissociative identity disorder ranging from 58% to 90% (Brand et al., 2009; Coons, 1994; Ellason, Ross, and Fuchs, 1996). Furthermore, in the current PTSD sample with multiple exposures to different traumatic event types, 56% reported having experienced sexual assaults, whereas 46% reported at least one sexual assault with vaginal, anal, or oral penetration. In contrast, 17%, that is, a significantly smaller portion of the sample of patients with psychotic disorders, reported sexual assaults. This observation is consistent with the defense cascade model: traumatic event types with a high likelihood of violation, invasion, penetration, contamination, and similar dangers correspond with shutdown behavior. Additionally, correlates suggest that dissociation is closely related to PTSD symptoms. Whereas PTSD itself reflects a disorder that may already arise from the initial stages of the defense cascade (sympathetic arousal, panic, intrusions, hypervigilance, exaggerated startle responses, and irritability), shutdown dissociation reflects the more disparate and ultimate part of the response repertoire typical for complex trauma survivors (derealization, depersonalization, emotional numbing, analgesia, lack of visual intrusions, personality changes, and stuporous conditions). Taken together, the correlates support that shutdown dissociation, described in DSM-5 as PTSD with dissociative symptoms, develops in response to repeated exposure to traumatic stressors, especially those that include a high proximity to danger, usually alongside the core PTSD symptoms. The psychopathological and phenomenological relationship is further supported by the evidence that symptom reduction through exposure-based treatment may be lower for patients with dissociative symptoms (Jaycox, Foa, & Morral, 1998). Dissociation can pose a significant challenge to the successful implementation of exposure therapy for PTSD because it serves to escape intense emotions and is likely to interfere with information processing (Harned, 2013). Exposure treatment for dissociative patients may unmask the PTSD symptoms (Hagenaars, Van Minnen, & Hoogduin, 2010). Hence, trauma survivors with shutdown dissociation require different treatment strategies as well as psychoeducation regarding shutdown dissociation and handling in therapy. A patient with a tendency towards shutdown dissociation could respond to exposure therapy with a functional sensory deafferentiation, motor paralysis, loss of language production and understanding, emotional numbing, and parasympathetic-driven physiological responses such as bradycardia, reduction of blood pressure, and, in the worst case, vasovagal fainting (Schauer & Elbert 2010). These heavy clinical states of shutdown dissociation could discourage therapists from performing exposure therapy (Hembree & Cahill, 2007). The Shut-D allows for the assessment of the severity and expression of the patients’ shutdown symptoms, which enables the therapist to prepare him or herself for applying anti-dissociative strategies and physical counter maneuvers during the exposure session (Schauer & Elbert, 2010). Conveying the biological underlying principles for dissociative behavior in humans, the Shut-D may furthermore help clear the way for a synopsis that unifies the different concepts of “dissociation.” The Shut-D will foster greater awareness and help to systematically record the impact of traumatic experiences with a high proximity to danger, such as sexual assault. In addition, the Shut-D can assess the phenomenon underlying the dissociative subtype of PTSD described in DSM-5.

## Limitations and conclusion

The inter-rater reliability and interviewer bias have not been systematically assessed. Furthermore, the data used for the psychometric characterization of PTSD patients were exclusively gathered from women. We focused on women rather than men because women are more likely to cover the full range (low dissociative to high dissociative) of shutdown dissociation. Anecdotal reports as well as our own clinical experience also suggest the presence of shutdown dissociation in men (Noyes & Kletti, 1977). A reason for the sex difference may be that sexual assault, a traumatic event type that coincides with maximum proximity to danger, is more likely to strike women than men. The diagnoses of patients from the study samples 2 and 3 were obtained by the responsible psychologist/psychiatrist and were not further validated by independent expert ratings.

At present, the Shut-D may serve as a useful tool for clinicians who apply exposure-based treatment and as a research instrument for assessing shutdown dissociation based on the defense cascade model. The high correlation with the DES adds to the construct validity of the scale. Further studies are necessary to establish discriminant validity as well as differential aspects of the scale. However, the item construction differs from other measure of dissociation following the biological and neural system rather than phenomenological compilations. To what extent this newly developed scale proves to be more effective and useful for etiological or Research Domain Criteria-based dimensional modeling than previous scales should be subject to further research questions. The psychometric properties justify the assessment of shutdown dissociative responding following traumatic experiences (with different proximity to danger), and the awareness of shutdown dissociation offers innovation and improvement in treatment strategies.

## Footnotes

1 With the kind permission of the Institute of Psychology, University of Zurich, Switzerland (Yolanda Schlumpf, Ekaterina Weder, and Prof. Lutz Jäncke).
